# Characterization of Virulence Phenotypes of *Heterodera glycines* during 2020 in Indiana

**DOI:** 10.2478/jofnem-2023-0039

**Published:** 2023-10-04

**Authors:** Ricky Critchfield, Jaden King, John Bonkowski, Darcy Telenko, Tom Creswell, Lei Zhang

**Affiliations:** Department of Botany and Plant Pathology, Purdue University, West Lafayette, IN 47907; Department of Entomology, Purdue University, West Lafayette, IN 47907

**Keywords:** soybean cyst nematode, virulence phenotype, HG type, soybean, nematode management

## Abstract

The soybean cyst nematode (SCN, *Heterodera glycines*) is the most yield-limiting pathogen of soybean in the US. This study was carried out in order to provide updated information on SCN virulence phenotypes in Indiana. A total of 124 soil samples were collected from soybean fields in 2020 and all of them tested positive for SCN. The virulence phenotypes of 42 representative SCN populations were determined with seven soybean indicator lines using the standard HG type test. The most predominant HG types were 2.5.7 and 1.2.5.7, which accounted for 64% and 14% of the SCN populations tested, respectively. None of the SCN populations tested were rated as HG type 0, compared with 28% of the populations in a previous survey in Indiana during 2006–2008. Nearly 88% of the SCN populations evaluated in this study overcame the resistance provided by PI 88788, which is the most common source of resistance in soybean, up from 56% in the 2006–2008 survey. Approximately 14% of SCN populations tested were virulent to PI 548402 (Peking), in contrast to 0% in the 2006–2008 survey. This study reveals a trend of increasing virulence of SCN populations to resistant sources of soybean in Indiana. The results highlighted the importance of rotating soybean varieties with different types of resistance and identifying new sources of resistance for sustainable management of SCN.

Soybean (*Glycine max* (L.) Merr.) is one of the most important leguminous crops globally, providing protein and oil for humans and livestock. In Indiana, over 5 million acres of soybeans were planted and harvested in 2020 (USDA-NASS 2020). The soybean cyst nematode (SCN), *Heterodera glycines* Ichinohe, is the most damaging pathogen of soybean. The soybean cyst nematode is an obligate sedentary endoparasitic cyst nematode that infects soybean roots and forms a feeding site called a syncytium to obtain nutrients from soybean and complete its life cycle (Mitchum, 2016). The annual soybean yield losses caused by SCN are estimated at more than $1.5 billion in the United States (Bandara et al., 2020). However, losses caused by SCN usually go unnoticed because SCN can cause yield loss of up to 30% without any visible aboveground symptoms (Wang et al., 2003).

After its initial discovery in North Carolina in 1954, SCN has been reported in every soybean-producing state in the United States except for West Virginia (Tylka and Marett, 2021; Winstead et al., 1955). The two primary methods of SCN management have been host resistance and non-host crop rotation (Nissan et al., 2022). Although many different soybean varieties resistant against SCN are available in the United States, more than 95% of the resistant varieties derive their resistance from a single genetic source, PI 88788 (McCarville et al., 2017). Repeated use of soybean varieties with the same source of resistance can select for SCN virulent populations that can overcome and reproduce on resistant varieties, making the host resistance less effective at controlling SCN (Bent, 2022; Colgrove and Niblack, 2008). Therefore, knowledge of SCN virulence phenotypes is important to devise management recommendations and guide efforts to breed soybean varieties with new sources of resistance.

A classification system known as “HG type” was developed to characterize the virulence phenotypes of SCN populations. The HG stands for the scientific name of the soybean cyst nematode, *Heterodera glycines* (Niblack et al., 2002). The HG type test involves testing the reproduction of SCN populations on seven soybean indicator lines with resistance to SCN relative to a test of susceptible soybeans. These resistant lines are PI 548402 (‘Peking’, #1), PI 88788 (#2), PI 90763 (#3), PI 437654 (#4), PI 209332 (#5), PI 89772 (#6) and PI 548316 (#7). The virulence phenotype of a given SCN population is determined by assessing its relative reproductive potential on these seven different resistant indicator lines compared to a susceptible soybean check. The result is expressed as the female index (FI). An FI ≥ 10% on a given indicator line indicates that the SCN population is virulent and can reproduce significantly on the indicator line (Niblack et al., 2002).

Several reports using the HG type test have indicated that the commonly used resistance source PI 88788 is being overcome in different soybean growing states such as Minnesota (Zheng et al., 2006), Illinois (Faghihi et al., 2010; Kleczewski et al., 2022; Niblack et al., 2008), Iowa (McCarville et al., 2017), Tennessee (Faghihi et al., 2010), North Dakota (Chowdhury et al., 2021) and South Dakota (Acharya et al., 2016). Most importantly, two surveys of SCN virulence phenotypes in Missouri in 2005 and 2015–2016 indicated that it was increasing, especially on PI 88788 and Peking, the most commonly used two sources of resistance to SCN (Howland et al., 2018; Mitchum et al., 2007).

The soybean cyst nematode was first identified in Indiana in 1973 and has spread to the entire state except for Monroe and Brown counties (Faghihi et al., 1986; Tylka and Marett, 2021). The Extension Nematology program at Purdue led by Drs. Virginia Ferris and Jamal Faghihi provided nematode diagnostic services, monitored SCN distribution in Indiana, and developed the soybean line CystX with broad-spectrum resistance against various SCN populations for effective SCN management (Faghihi, 2017). The last SCN virulence survey in Indiana was conducted by the program in 2006–2008, more than a decade ago (Faghihi et al., 2010). Therefore, the objective of this study was to determine and provide an updated profile of SCN virulence phenotypes in Indiana.

## Materials and Methods

*Soil samples and nematode extractions:* To obtain soil samples for this study, we requested that soybean growers in Indiana send us soil samples collected on their fields. A total of 124 soil samples were submitted by soybean growers from 32 different counties in Indiana in 2020 (Supplementary Table 1). The request for soil samples from soybean fields was sent to soybean growers of Indiana via the Pest & Crop Newsletter of Purdue University and Morning AgClips News in 2020. Soybean growers were asked to follow Purdue protocols of collecting 10 to 20 soil cores in a zig-zag pattern in fields, with each soil core 1 inch in diameter and 6 to 8 inches in depth, until 500 cm^3^ soil had been collected from several areas of a field (Faghihi et al., 2010). The soil samples were placed in plastic bags and stored at 4°C until processing for extraction of cysts and eggs. Each soil sample was thoroughly mixed before being subsampled for cyst extraction (250 cm^3^ of soil per subsample) with a modified method of sieving and decanting procedure (Krusberg et al., 1994). Each soil subsample was suspended in tap water by stirring in a bucket. Ten seconds after stopping stirring, the supernatant, containing cysts, was poured through a stack of sieves of 850- and 125-μm apertures (VWR, PA, USA), with the top sieve (850-μm) to remove larger particles and residual root tissue. Cysts and any smaller debris collected on the bottom sieve (125-μm) were then transferred with 40 ml of water into a 50-mL centrifuge tube and centrifuged at 3000 rpm for 4 minutes to get the cysts to the bottom. After removing the supernatant, the cyst-soil pellet was resuspended in 40 ml of 70% sucrose solution and centrifugated at 3000 rpm for 4 minutes. Cysts in the supernatant were then collected with a 125-μm sieve and rinsed with tap water to remove sucrose. Two more sieves (45-, and 25-μm) were then put under the 125-μm sieve. Cysts on the 125-μm sieve were crushed manually using a rubber stopper and rinsed with tap water frequently to release eggs (Faghihi and Ferris, 2000). The eggs and second-stage juveniles collected on the 25-μm sieve and were collected and counted with a ZEISS Stemi 508 stereo-microscope (Zeiss, White Plains, NY).

*HG type test:* The SCN populations for the HG type test were selected based on their county locations in Indiana. We performed simple random sampling using the “sample” function in R to select the SCN populations within each county included in this study. To obtain sufficient inoculum for the HG type test, eggs extracted from selected soil samples were used to inoculate the susceptible soybean variety Williams 82 for 1 to 2 months to increase their populations. The increase of SCN field populations on Williams 82 for a couple of generations may reduce the dormant SCN eggs and also other contaminated parasites, which could otherwise affect the HG type test (Niblack et al., 2009). Williams 82 was used as the susceptible check in the HG type test, because it has similar susceptibility as Lee 74, the standard cultivar used as a susceptible check for the HG type test, but Williams 82 seeds germinate significantly better than those of Lee 74 (Niblack et al., 2009).

The indicator lines PI 548402 (#1, ‘Peking’), PI 88788 (#2), PI 90763 (#3), PI 437654 (#4), PI 209332 (#5), PI 89772 (#6), PI 548316 (#7), PI 567516C, and Williams 82 (the susceptible check) were used to determine SCN population HG type (Niblack et al., 2002). The soybean seeds were germinated in Cone-tainers (Stuewe & Sons, OR, USA) containing 3:1 thoroughly mixed sand and soil mixture. The seeds were germinated directly in the mixture for 10 days. Five plants (one plant per cone-tainer) of each soybean line were used for SCN inoculation. 3500 SCN eggs and juveniles were used to inoculate each plant. The soybean plants were completely randomized and maintained in a growth chamber for 35 days after inoculation, with a temperature of 24–26°C and a photoperiod of 16-hr-light and 8-hr-dark (Niblack et al., 2002; Niblack et al., 2009). After 35 days post-inoculation, plants were taken down and SCN females were extracted from both roots and soil. The numbers of females were counted under a stereo-microscope and female index (FI) was calculated as follows: (average number of females on an indicator line/average number of females on the susceptible check of Williams 82) × 100.

*Data analysis:* The average number of SCN females from 5 replicates per indicator line was used to determine the FI and the HG type of each SCN population. The HG type was determined by assaying which indicator lines had an FI ≥ 10%. Microsoft Excel (Microsoft, Redmond, Washington) was used for the descriptive statistics of the FI datasets. R (version 4.2.1, R Foundation for Statistical Computing, Vienna, Austria) and RStudio (version 2022.07.2+576 “Spotted Wakerobin”, Posit Software, Boston, MA) were used to conduct Pearson's product moment correlation analysis for the seven indicator lines and PI 567516C.

## Results

**Figure 1:**
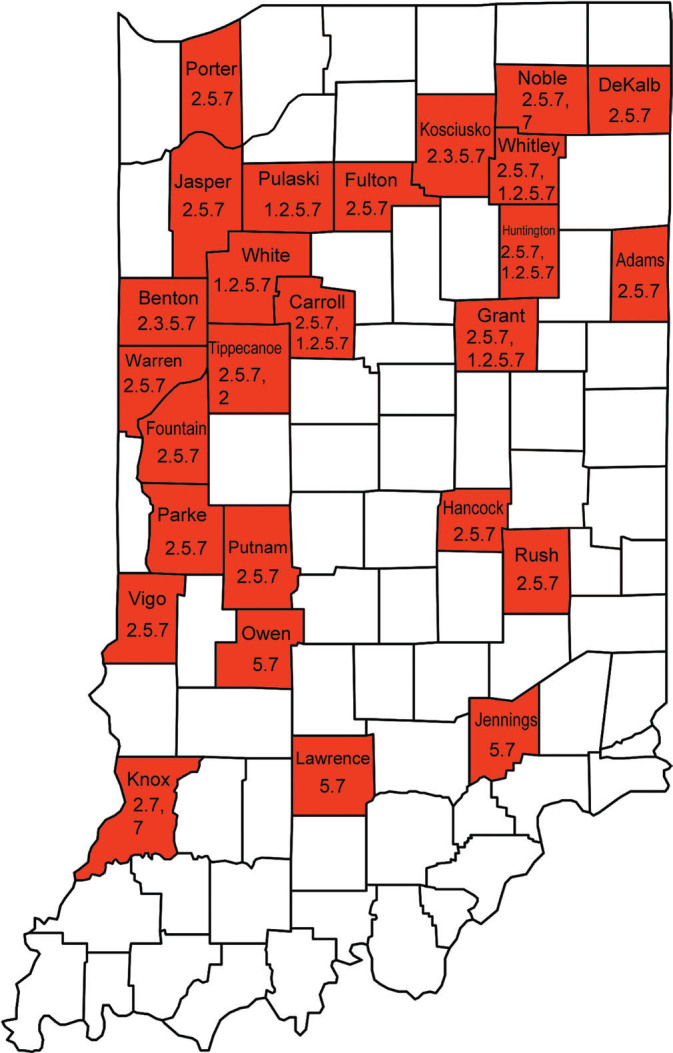
A map of counties in Indiana where *Heterodera glycines* virulence phenotypes were tested using soil samples collected in 2020. The *H. glycines* virulence type(s) found in each county tested are labeled on the map.

To provide updated information on SCN virulence phenotypes in Indiana, a total of 124 soil samples were collected from soybean fields in the state in 2020. All of the soil samples (100%) were tested positive for SCN. The SCN population densities of the samples ranged from 165 to 19,840 eggs and juveniles per 100 cm^3^ of soil. The soil sample with the highest SCN population density was collected from a field in Noble County, and the soil sample with the lowest population density was from Tippecanoe County (Supplementary Table 1). The average population density of all the samples was 3,207 eggs and juveniles per 100 cm^3^ of soil. Among the 124 samples, 92 (74.2%) had a population density greater than 500 eggs and juveniles per 100 cm^3^ of soil. Forty-two SCN populations from 26 counties in Indiana were selected for the SCN HG type test using Williams 82 as the susceptible check (Table 1 and Fig. 1) (Niblack et al., 2002; Niblack et al., 2009). In addition to the 7 classic soybean indicator lines, the SCN-resistant line PI 567516C was included to test its resistance against the SCN populations collected in this study, although it is not an official indicator line in the SCN HG type test. PI 567516C showed different levels of resistance to multiple SCN HG types and its genetic resistance to SCN conferred by two QTLs was different from the major sources of resistance, including PI 88788 and Peking (Arelli et al., 2009; Usovsky et al., 2021; Vuong et al., 2010; Zhuang et al., 2022).

**Table 1.** *Heterodera glycines* (HG) virulence phenotypes detected from soil samples collected in Indiana in 2020

**Sample ID**	**County**	**Female index (FI)^a^**										**Mean^b^**	**HG Type**

		**PI 548402 #1**	**PI 88788 #2**	**PI 90763 #3**	**PI 437654 #4**	**PI 209332 #5**	**PI 89772 #6**	**PI 548316 #7**	**PI 567516C**				
48	Adams	7.6	34.2	2.6	1.5	26.4	1.4	37.6	0.0	217	2.5.7
44	Benton	0.4	30.1	15.6	0.0	49.2	0.0	42.3	0.0	666	2.3.5.7
82	Carroll	1.5	73.5	0.2	0.0	44.6	0.2	28.0	14.4	249	2.5.7^c^
83	Carroll	19.2	64.3	0.4	0.0	70.4	0.0	84.4	9.4	110	1.2.5.7
96	Carroll	2.4	84.8	0.5	1.3	75.7	1.5	80.0	4.4	168	2.5.7
4	DeKalb	0.5	70.5	0.0	0.0	46.6	0.3	57.0	9.6	223	2.5.7
34	DeKalb	4.7	26.5	6.0	6.8	26.0	3.3	51.5	3.1	262	2.5.7
12	Fountain	0.6	37.5	0.1	0.3	25.4	0.5	32.3	5.7	236	2.5.7
69	Fulton	0.7	62.8	0.3	0.0	58.5	0.8	69.6	0.0	371	2.5.7
33	Grant	2.1	12.5	0.5	0.4	19.1	0.4	22.4	12.7	572	2.5.7^c^
50	Grant	0.0	23.7	0.0	0.0	38.0	0.0	27.7	11.8	102	2.5.7^c^
51	Grant	16.8	65.2	1.0	1.1	57.4	0.1	57.9	7.5	306	1.2.5.7
92	Hancock	2.1	32.2	0.7	0.0	23.4	0.1	40.8	1.9	407	2.5.7
18	Huntington	2.1	21.0	0.6	3.0	22.3	0.8	68.0	0.3	488	2.5.7
21	Huntington	12.6	23.7	3.0	0.0	34.0	4.5	65.4	26.0	170	1.2.5.7^c^
27	Huntington	0.9	24.1	1.1	1.1	33.0	0.7	12.3	8.7	68^*^	2.5.7
29	Huntington	5.4	24.4	0.0	0.2	34.9	0.0	62.3	11.9	319	2.5.7^c^
30	Huntington	0.8	15.7	0.1	0.0	15.8	0.1	47.3	3.0	527	2.5.7
6	Jasper	4.4	64.0	0.9	1.7	35.4	0.8	42.5	8.2	93^*^	2.5.7
117	Jennings	0.6	8.5	0.0	0.0	16.0	0.0	12.1	18.8	94^*^	5.7^c^
78	Knox	1.8	11.4	0.5	1.2	1.2	1.5	22.3	20.5	178	2.7^c^
85	Knox	0.0	6.7	0.0	0.3	5.6	0.0	16.9	0.0	142	7
77	Kosciusko	6.2	70.6	20.3	3.7	43.6	2.0	15.2	2.9	399	2.3.5.7
118	Lawrence	0.7	3.5	0.0	0.0	12.6	0.0	15.3	2.6	228	5.7
108	Noble	2.2	50.4	0.4	0.0	73.7	0.0	33.7	1.6	179	2.5.7
109	Noble	3.1	16.9	0.3	0.1	47.2	0.4	49.3	7.7	681	2.5.7
110	Noble	1.4	5.8	2.0	0.6	4.3	0.4	14.5	2.9	325	7
112	Noble	2.5	58.0	0.5	1.6	24.7	0.0	16.9	0.9	95^*^	2.5.7
121	Owen	2.3	3.9	0.4	0.0	14.1	0.0	25.5	3.8	331	5.7
11	Parke	1.1	16.1	0.2	1.1	17.6	0.4	24.3	0.0	602	2.5.7
104	Porter	1.6	30.2	0.9	0.3	46.5	0.0	66.1	5.9	291	2.5.7
123	Porter	1.0	77.6	1.9	0.0	69.1	0.3	72.3	37.7	180	2.5.7^c^
54	Pulaski	13.0	46.5	4.7	0.0	35.6	2.6	47.7	2.1	80^*^	1.2.5.7
9	Putnam	6.7	70.5	3.8	2.1	76.2	6.6	62.3	0.0	126	2.5.7
88	Rush	2.5	15.3	0.1	0.1	20.3	0.3	24.1	3.6	872	2.5.7
61	Tippecanoe	1.9	17.6	0.9	1.3	3.8	0.2	9.0	1.2	511	2
71	Tippecanoe	0.3	80.8	0.2	0.1	45.6	0.0	63.1	3.1	243	2.5.7
56	Vigo	5.1	62.7	0.0	0.0	57.5	9.1	78.1	5.7	213	2.5.7
115	Warren	5.2	35.9	0.9	0.0	33.9	0.0	34.8	3.7	216	2.5.7
80	White	38.1	69.1	0.0	0.0	61.8	5.4	59.3	1.9	179	1.2.5.7
53	Whitley	18.4	72.5	0.0	0.0	42.6	6.1	74.4	2.3	165	1.2.5.7
94	Whitley	1.5	36.6	0.2	0.3	17.5	0.0	31.0	5.8	557	2.5.7

a FI = (average number of females on an indicator line/average number of females on the susceptible check Williams 82) × 100.

b Average number of females on the susceptible check Williams 82. SCN populations with an average number of females on Williams 82 less than 100 were indicated with ^*^.

c SCN population that had an FI >10% on PI 567516C.

Out of the 42 SCN populations tested, six populations (14.3%) that originated from Carroll, Grant, Huntington, Pulaski, White, Whitley counties were capable of reproducing on PI 548402 (HG type indicator line #1, ‘Peking’) with FI ≥ 10% (Tables 1 and 2). The FI values of these six populations ranged from 12.6% to 38.1%, with an average FI of 19.7% (Table 2). The SCN population with the highest FI value on PI 548402 was collected from White County (Table 1).

**Table 2.** Univariate descriptive statistics of female indices (FI) of soybean cyst nematode populations that produced an FI ≥ 10% on soybean indicator lines

**Soybean Indicator Line**	**FI value (%)^b^**			

	**FI ≥ 10%^a^**	**Minimum Observed**	**Maximum Observed**	**Average**
PI 548402 (#1)	14.3	12.6	38.1	19.7
PI 88788 (#2)	88.1	11.4	84.8	43.9
PI 90763 (#3)	4.8	20.3	25.5	22.9
PI 209332 (#5)	90.5	12.6	76.2	39.8
PI 548316 (#7)	97.6	12.1	84.4	43.8
PI 567516C	19.0	11.8	37.7	19.2

a Percentage of SCN populations with an FI ≥ 10%.

b Only FI ≥ 10% were included.

On PI 88788 (HG type indicator line #2), 37 out of 42 HG-typed populations (88.1%) reproduced with an FI ≥ 10% (Tables 1 and 2). The FI values among these SCN populations ranged from 11.4% to 84.8%, with an average FI of 43.9% (Table 2). Twenty-four out of these 37 populations (64.9%) had an FI ≥ 30% on PI 88788, but 16 (43.2%) had an FI ≥ 50% on PI 88788 (Table 1). These 37 SCN populations originated from 23 of the 26 counties in Indiana where soil samples were assayed (Table 1). The SCN population with the highest FI value (84.8%) on PI 88788 was collected from Carroll County (Table 1).

For the soybean line PI 209332 (HG type indicator line #5), 90.5% of the SCN populations (38 out of 42) tested had an FI ≥ 10% (Table 2). The SCN population with the highest FI value (FI = 76.2%) on PI 209332 originated from Putnam County. The rest of the SCN pullulations that parasitized on PI 209332 had FI ranging from 12.6% to 75.7% (Tables 1 and 2). Overall, 24 of these 39 populations (61.5%) had an FI ≥ 30% and 9 (23.1%) had a FI ≥ 50% on PI 209332.

The soybean line PI 548316 (HG type indicator line #7) had the highest number of SCN populations with an FI ≥ 10%. All but one of the 42 (97.6%) SCN populations tested had an FI ≥ 10% on PI 548316 (Tables 1 and 2). The FI values of the populations virulent on PI 548316 ranged from 12.1% to 84.4%, with an average FI of 43.8% (Table 2). The SCN population with the highest FI value on PI 548316 was collected from Carroll County. Overall, 27 among these 41 populations (65.9%) had a FI ≥ 30% and 16 (39%) had a FI ≥ 50% (Table 1).

Only two of the SCN populations tested had an FI ≥ 10% on PI 90763 (HG type indicator line #3) with FI = 15.6% and 20.3%, and they were from Benton County and Kosciusko County, respectively (Table 1). None of the SCN populations tested had an FI ≥ 10% on PI 437654 (HG type indicator line #4) or PI 89772 (#6). The highest FI value on PI 437654 was 6.8% and the greatest FI value on PI 89772 was 9.1%. For the resistant line PI 567516C included in this study, 8 out of the 42 SCN populations (19%) were capable of reproducing on it with an FI ≥ 10%, ranging from 11.8% to 37.7% (Table 2).

In total, seven different HG types were detected among SCN populations tested in this study: HG type 2.5.7, 1.2.5.7, 5.7, 2.3.5.7, 2.7, 7, and 2. The most commonly found SCN virulence phenotype was HG type 2.5.7 (Table 3). Correlation analysis was conducted on the FI values of different soybean indicator lines. The FI values of the indicator lines PI 88788 (#2), PI 209332 (#5), and PI 548316 (#7) were positively correlated (*P* < 0.01) with each other. PI 548402 (#1) was positively correlated with PI 88788 (#2), PI 209332 (#5) (*P* < 0.05) and PI 89772 (#6), PI 548316 (#7) (*P* < 0.01) (Table 4). No significant correlation was observed for PI 567516C with the seven indicator lines. We also did not find significant correlation for PI 90763 (#3) and PI 437654 (#4) with other indicator lines or PI 567516C (Table 4).

**Table 3.** Frequencies of HG types in soil samples collected in 2020 in Indiana

**Type**	**No. of Populations^a^**	**Frequency (%)**
2.5.7	27	64.3
1.2.5.7	6	14.3
5.7	3	7.1
2.3.5.7	2	4.8
7	2	4.8
2.7	1	2.4
2	1	2.4

a Number of soybean cyst nematode populations (out of 42 tested in this study).

**Table 4.** Correlation coefficient among soybean indicator lines with resistance to soybean cyst nematode (SCN) based on female indices (FI)^a^ from 42 SCN field populations collected in 2020 in Indiana

**Indicator Line**	**PI 548402**	**PI 88788**	**PI 90763**	**PI 437654**	**PI 209332**	**PI 89772**	**PI 548316**
PI 88788	0.360^*^						
PI 90763	−0.022 ns	0.057 ns					
PI 437654	−0.037 ns	0.028 ns	0.303 ns				
PI 209332	0.345^*^	0.799^**^	0.120 ns	−0.100 ns			
PI 89772	0.501^**^	0.342^*^	0.051 ns	0.169 ns	0.311^*^		
PI 548316	0.396^**^	0.591^**^	−0.091 ns	−0.048 ns	0.675^**^	0.431^**^	
PI 567516C	−0.050 ns	0.045 ns	−0.140 ns	−0.202 ns	0.111 ns	−0.051 ns	0.114 ns

a FI = (average number of females on an indicator line/average number of females on the susceptible check Williams 82) × 100.

* *P* < 0.05;

** *P* < 0.01

ns: not significant (*P* > 0.05)

## Discussion

Monitoring SCN virulence phenotypes is critical for establishing management recommendations for soybean growers and guiding soybean breeding efforts (Howland et al., 2018; Niblack et al., 2002). In this study we asked soybean growers in Indiana to send us soil samples, and in return we provided HG type information and management recommendations to growers. This approach can provide field-specific information on virulence types of SCN populations directly to growers and raise their awareness of the presence of virulent SCN populations in their fields.

Different approaches had been employed by previous studies to collect soil samples for surveying the SCN virulence phenotypes, depending on the available resources. For example, the last similar study conducted in Indiana during 2006–2008 used soil samples submitted by growers to the Purdue Nematode Diagnostic Lab (Faghihi et al., 2010). A recent SCN virulence survey in Illinois collected soil samples by collaborating with extension professionals and researchers (Kleczewski et al., 2022).

In this study, a total of seven different HG types were detected in 42 SCN field populations collected in 2020 in Indiana. The 42 SCN samples were from 26 counties in Indiana (Table 1 and Fig. 1). When more than one sample in the same county was included in the HG type test, we randomly selected the soil samples. Of these 26 counties, five counties (Benton, Grant, Knox, Rush, and White) were among the top 10 counties in terms of the harvested acres of soybean in 2020 in Indiana (USDA-NASS, 2020). These 26 counties, out of 92 counties in Indiana, accounted for more than 35% of the total harvested acres of soybean in 2020 (USDA-NASS, 2020). The counties in the south central and southeast regions of Indiana, which were not well covered by the submitted soil samples in this study, usually had fewer harvested acres of soybean; 33% of the counties in these two regions had fewer than 10,000 harvested acres per county in 2020.

The HG types of SCN populations from the 26 counties found in this study provided insight into the overall trends of SCN virulence in Indiana in 2020. The most predominant SCN virulence phenotype was HG type 2.5.7. Out of the 42 SCN populations tested, 64.3% were HG type 2.5.7, followed by HG type 1.2.5.7 (14.3%), HG type 5.7 (7.1%), HG type 2.3.5.7 (4.8%), HG type 7 (4.8%), HG type 2.7 (2.4%) and HG type 2 (2.4%). Similar patterns of SCN virulence phenotypes were observed in 52 SCN populations in Illinois and 48 in Missouri, where the HG type 2.5.7 was prevalent (Howland et al., 2018; Kleczewski et al., 2022).

In contrast, HG type 7 was the predominant SCN virulence phenotype in North Dakota, South Dakota and Minnesota (Acharya et al., 2016; Chowdhury et al., 2021; Zheng et al., 2006). The differences in regional patterns of SCN virulence phenotypes may be related to soil and environmental conditions, as well as cropping practices and the soybean varieties planted.

Our study of SCN populations collected in 2020 revealed a significant shift in SCN virulence phenotypes in Indiana compared to the last survey conducted during 2006–2008 (Faghihi et al., 2010). The soil samples of the 2006–2008 survey were submitted by soybean growers to the Purdue Nematode Diagnostic Laboratory, which was closed in 2018. HG type 0 accounted for 28% of the SCN populations tested in 2006–2008, but was not detected at all in our survey. A recent survey in Missouri conducted in 2015–2016 did not detect HG type 0 either (Howland et al., 2018). HG type 0 indicates that an SCN population cannot reproduce on any of the 7 indicator lines (with FI < 10%). Absence of HG type 0 populations in our survey indicated that more SCN field populations in Indiana have become virulent on these soybean indicator lines, which are the main sources for breeding resistance against SCN.

The most prevalent SCN virulence type in the 2006–2008 survey was HG type 2.5.7. However, only 34.2% of the 2006–2008 SCN populations were HG type 2.5.7, indicating that there had been a significant increase by the time of the 2020 survey (64.3%). Furthermore, more than 88% of SCN populations tested in our study had an FI ≥ 10% on PI 88788 (indicator line #2), compared to 56% in 2006–2008 (Faghihi et al., 2010). The significant increase in the percentage of Indiana SCN populations with an FI ≥ 10% on PI 88788 was expected, as it follows similar HG type shifts of SCN populations in other states. The majority of SCN-resistant varieties available to north central regions of United States were also derived from the PI 88788 (Howland et al., 2018; Kleczewski et al., 2022; McCarville et al., 2017; Mitchum et al., 2007; Niblack et al., 2008). Since increased SCN virulence on PI 88788 can be directly correlated with yield losses in soybean varieties with PI 88788-type resistance, the higher percentage of SCN populations virulent on PI 88788 (FI ≥ 10%) may have significantly reduced the soybean yield potentials in Indiana (McCarville et al., 2017). A statistically significant positive correlation was detected among the FI values of the soybean indicator lines #2, #5 and #7 (Table 4), probably because the indicator lines PI 209332 (#5) and PI 548316 (#7) were reported to have the PI 88788-type resistance, as well as utilizing the *Rhg1-b* resistance allele to confer resistance against SCN (Colgrove and Niblack 2008; Cook et al., 2012). Therefore, soybean breeding efforts need to avoid using resistance sources from PI 209332 (#5) and PI 548316 (#7) because SCN populations that are able to reproduce on PI 88788 (#2) tend to overcome the resistance from soybean lines #5 and #7.

None of the SCN populations in the 2006–2008 survey of Indiana had an FI ≥ 10% on PI 548402 (#1, Peking). In this study, 14.3% of the SCN field populations tested showed significant reproduction on PI 548402 (FI ranging from 12.6% to 38.1%) and were all determined to be HG type 1.2.5.7. This is the first time that SCN populations virulent against PI 548402 (Peking) have been reported in Indiana. Although PI 88788-type and Peking-type resistances are different, a positive correlation between the FI values of the indicator line #1 (Peking) and #2 (PI 88788) was detected in this study. Similar correlations between #1 and #2 were also reported in previous SCN surveys conducted in North Dakota and Missouri (Chowdhury et al., 2021; Niblack et al., 2003). Our data indicated that soybean growers in Indiana need to monitor SCN virulence types in their fields when they consider using varieties with resistance derived from PI 548402 (Peking).

None of the SCN populations studied in the 2006–2008 survey had an FI ≥ 10% on PI 90763 (#3) (Faghihi et al., 2010). In this study, we detected only two SCN populations with an FI ≥ 10% on PI 90763; both are HG type 2.3.5.7 and were from Benton and Kosciusko Counties in Indiana. Similar to the 2006–2008 survey, our study did not detect SCN populations that had an FI ≥ 10% on PI 437654 (#4) or PI 89772 (#6). Most of the FI values on #4 and #6 were close to 0%, with the highest FI values being 6.8% on #4 and 9.1% on #6. Our data indicated that sources of resistance from PI 90763 (#3), PI 437654 (#4) and PI 89772 (#6) are still effective against most of the virulent SCN populations in Indiana.

PI 567516C has a unique, though not well elucidated, mechanism of resistance, and does not rely on *Rhg1-b* or *Rhg4* (Vuong et al., 2010). The currently known QTLs responsible for resistance to SCN in PI 567516C are qSCN10 and qSCN18 (Usovsky et al., 2021; Zhou et al., 2021). PI 567516C was included this survey in an effort to test the virulence of the SCN populations of Indiana on this resistant line. We found that eight SCN populations (19% of the 42 populations) had an FI ≥ 10% on PI 567516C. Therefore, although it is a promising new source of genetic resistance against SCN, PI 567516C may need to be used together with other sources of resistance for sustainable SCN control. Overall, it is necessary to monitor SCN population densities and virulence phenotypes on soybean fields for effective and sustainable control of SCN using different management tactics, including rotations using soybean varieties with different sources of resistance or with non-host crops.

## Supplementary Material

Supplementary Material Details

## Figures and Tables

**Supplementary Table 1:** Population densities of soybean cyst nematode in soil samples collected in Indiana in 2020

**County**	**Number of soil samples^a^**	**Eggs and Juveniles**		

		**Minimum No.^b^**	**Maximum No.^c^**	**Average No.^d^**
Adams	2	2500	5505	4003
Allen	3	1370	2500	1940
Benton	7	300	4300	2098
Carroll	7	665	13308	5254
Clinton	1	600	600	600
DeKalb	6	240	15600	4782
Fayette	4	400	10950	5316
Fountain	2	4950	10650	7800
Fulton	2	3320	6050	4685
Grant	4	400	6650	3358
Hancock	2	783	1050	916
Henry	2	417	483	450
Huntington	16	240	7265	1805
Jasper	4	220	17032	5669
Jennings	2	290	530	410
Knox	5	420	2100	1129
Kosciusko	1	10043	10043	10043
LaPorte	3	356	2280	1026
Lawrence	2	400	665	532
Montgomery	2	940	2610	1775
Noble	7	1020	19840	8313
Owen	2	290	300	295
Parke	1	2075	2075	2075
Porter	4	312	5940	2278
Pulaski	2	9045	10330	9688
Putnam	3	2425	13285	8070
Rush	2	853	2020	1437
Tippecanoe	14	165	6647	1418
Vigo	1	2120	2120	2120
Warren	1	765	765	765
White	4	550	6040	2888
Whitley	6	400	7040	2163

a Total number of soil samples collected in a county.

b Minimum number of SCN eggs and juveniles per 100 cm^3^ of soil.

c Maximum number of SCN eggs and juveniles per 100 cm^3^ of soil.

d Average number of SCN eggs and juveniles per 100 cm^3^ of soil.
