# Supplementary material for: Characterization of Virulence Phenotypes of Heterodera glycines during 2020 in Indiana
Source: J Nematol. 2023 Oct 4;55(1):20230039. doi: 10.2478/jofnem-2023-0039 (PMC10577647; doi:10.2478/jofnem-2023-0039)
Supplement: Supplementary file 1 — Supplementary Material Details [file jofnem-2023-0039_sm.pdf]

Supplementary Table 1: Population densities of soybean cyst nematode in soil samples collected in Indiana in 2020

County	Number of soil samples[a]	Eggs and Juveniles		
		Minimum No.[b]	Maximum No.[c]	Average No.[d]
Adams	2	2500	5505	4003
Allen	3	1370	2500	1940
Benton	7	300	4300	2098
Carroll	7	665	13308	5254
Clinton	1	600	600	600
DeKalb	6	240	15600	4782
Fayette	4	400	10950	5316
Fountain	2	4950	10650	7800
Fulton	2	3320	6050	4685
Grant	4	400	6650	3358
Hancock	2	783	1050	916
Henry	2	417	483	450
Huntington	16	240	7265	1805
Jasper	4	220	17032	5669
Jennings	2	290	530	410
Knox	5	420	2100	1129
Kosciusko	1	10043	10043	10043
LaPorte	3	356	2280	1026
Lawrence	2	400	665	532
Montgomery	2	940	2610	1775
Noble	7	1020	19840	8313
Owen	2	290	300	295
Parke	1	2075	2075	2075
Porter	4	312	5940	2278
Pulaski	2	9045	10330	9688
Putnam	3	2425	13285	8070
Rush	2	853	2020	1437
Tippecanoe	14	165	6647	1418
Vigo	1	2120	2120	2120
Warren	1	765	765	765
White	4	550	6040	2888
Whitley	6	400	7040	2163

[a] Total number of soil samples collected in a county.

[b] Minimum number of SCN eggs and juveniles per 100 cm^3 of soil.

[c] Maximum number of SCN eggs and juveniles per 100 cm^3 of soil.

[d] Average number of SCN eggs and juveniles per 100 cm^3 of soil.
